# Ascorbic acid promotes a TGF*β*1‐induced myofibroblast phenotype switch

**DOI:** 10.14814/phy2.13324

**Published:** 2017-09-14

**Authors:** Bram Piersma, Olaf Y. Wouters, Saskia de Rond, Miriam Boersema, Rutger A. F. Gjaltema, Ruud A. Bank

**Affiliations:** ^1^ Department of Pathology and Medical Biology Matrix research Group University of Groningen University Medical Center Groningen Groningen The Netherlands; ^2^ Department of Pharmaceutical Technology and Biopharmacy University of Groningen Groningen The Netherlands

**Keywords:** Ascorbic acid, collagen, fibrosis, myofibroblast, TGF*β*1

## Abstract

l‐Ascorbic acid (AA), generally known as vitamin C, is a crucial cofactor for a variety of enzymes, including prolyl‐3‐hydroxylase (P3H), prolyl‐4‐hydroxylase (P4H), and lysyl hydroxylase (LH)‐mediated collagen maturation. Here, we investigated whether AA has additional functions in the regulation of the myofibroblast phenotype, besides its function in collagen biosynthesis. We found that AA positively influences TGF *β*1‐induced expression of *COL1A1*,*ACTA2*, and *COL4A1*. Moreover, we demonstrated that AA promotes *α* SMA stress fiber formation as well as the synthesis and deposition of collagens type I and IV. Additionally, AA amplified the contractile phenotype of the myofibroblasts, as seen by increased contraction of a 3D collagen lattice. Moreover, AA increased the expression of several TGF *β*1‐induced genes, including *DDR1* and *CCN2*. Finally, we demonstrated that the mechanism of AA action seems independent of Smad2/3 signaling.

## Introduction

l‐Ascorbic acid (AA), generally known as vitamin C, is a water‐soluble vitamin with antioxidant properties (Meister 1994). Unlike most animal species, humans are unable to synthesize AA due to a mutation in the enzyme l‐gulono‐1,4‐lactone oxidase and therefore depend on uptake from the diet (Drouin et al., 2011; Traber and Stevens 2011). The world's first clinical trial by James Lind revealed that AA supplementation through fruits and vegetables is an effective treatment for scurvy, a connective tissue disorder often found in sailors of the 17th and 18th century (Peterkofsky 1991; Traber and Stevens 2011). The antiscorbutic action of AA is ascribed to its function as cofactor for three enzyme families involved in the biosynthesis of collagens, namely prolyl‐3‐hydroxylases (P3H), prolyl‐4‐hydroxylases (P4H), and lysyl hydroxylases (LH) (Pinnell 1985). These *α*‐ketoglutarate‐dependent nonheme iron dioxygenases are responsible for the hydroxylation of proline and lysine residues in the assembly of the collagen triple helix (Russell et al. 1981; Hata and Senoo 1989; Saika et al. 1992; Gjaltema and Bank 2016). For these enzymes, AA acts as an electron donor in the catalytic cycle by reducing the highly reactive iron species (Fe^4+^ and Fe^3+^) into the catalytically active Fe^2+^ (Pinnell 1985; Traber and Stevens 2011). The formation of hydroxyproline (Hyp) is required for the stability of the triple helix (Berg and Prockop 1973; Jimenez et al. 1973); an unstable triple helix is prone to intracellular degradation (Barile et al. 1989; Ishida et al. 2009). Because of its role in collagen biosynthesis, AA has been implicated in the pathophysiology of fibrosis, a chronic pathology characterized by excessive extracellular matrix (ECM) accumulation and cross‐linking (Rockey et al. 2015).

It is known for a long time that AA is required as culture medium supplement for human fibroblasts (Hata and Senoo 1989; Guo et al. 2007), as human cells are unable to synthesize AA. However, many studies have overlooked this fact, which may have led to unreliable conclusions. For example, it has been shown that exposure of human fibroblasts to conditioned medium from fetal or adult stem cells results in decreased collagen levels when AA is present in the medium, showing an antifibrotic effect of stem cell‐conditioned medium (Mia and Bank 2015). In contrast, the opposite has been reported when AA is absent, leading to the conclusion that such conditioned medium is profibrotic (Kim et al. 2007; Ding et al. 2013). That the latter conclusion is incorrect, is illustrated by rodent fibroblasts that react to conditioned medium of stem cells in the same way as AA‐exposed human fibroblasts (Mao et al. 2003). This can be easily explained because, in contrast to human fibroblasts, rodent fibroblasts are able to synthesize AA themselves. This example shows that one should take care to provide the right additives into the culture medium when culturing human fibroblasts.

The myofibroblast is the key cell in the pathophysiology of fibrosis and it is specialized in the synthesis of ECM components, such as collagen (Hinz 2016). Chronic organ injury activates effector cells such as fibroblasts and pericytes to adopt a myofibroblast phenotype under the influence of the profibrotic cytokine transforming growth factor (TGF)*β*1 (Hao et al. 1999; Leask and Abraham 2004; Piersma et al. 2015). Here we show, by means of antibodies recognizing either procollagen, native collagen or denatured collagen the previously established effect of AA on regular collagen homeostasis. Furthermore, we investigated whether TGF*β*1 and AA act in synergy with respect to collagen deposition, and whether AA is involved in the TGF*β*1‐induced phenotype switch from fibroblasts to myofibroblasts. Our results indicate that AA works in synergy with actions of TGF*β*1 with respect to collagen deposition and – more unexpectedly – also in regulating a signature myofibroblast expression profile. We further showed that the involved mechanism of the latter is probably independent of canonical Smad signaling.

## Methods

### Cell culture

Human dermal fibroblasts were purchased from ATCC (CCD‐1093Sk [ATCC^®^ CRL‐2115^™^], Wesel, Germany), and subcultures were maintained in Eagle's minimal essential medium (EMEM, Lonza, Basel, Switzerland) supplemented with 2 mmol/L l‐glutamine, 1% penicillin/streptomycin (complete growth medium) and 10% heat‐inactivated fetal bovine serum (FBS). For all experiments, cells were seeded at 15,000 cells/cm^2^ in complete growth medium and left to adhere for 24 h before serum starvation. In brief, cells were starved in complete growth medium supplemented with 0.5% FBS (bare medium). After 18 h, cells were stimulated with either 0.17 mmol/L AA (A8960, l‐ascorbic acid 2‐phosphate sesquimagnesium hydrate; Sigma‐Aldrich, Zwijndrecht, the Netherlands), 10 ng/mL TGF*β*1 (100‐21C; PeproTech Ltd., London, United Kingdom), or both, for up to 6 days and medium was refreshed daily.

### RNA extraction and quantitative real‐time PCR

For gene expression analysis, total RNA was isolated at day 2 and day 6 using the Tissue Total RNA mini kit (Favorgen Biotech Corp., Taiwan). RNA quantity and purity were determined with UV spectrophotometry (NanoDrop Technologies, Wilmington, USA). RNA was reverse transcribed using the RevertAid First Strand cDNA synthesis kit (Thermo Fisher Scientific, Landsmeer, the Netherlands) according to manufacturer's instructions. Real‐time PCR was performed with SYBR green PCR master mix (Roche, Basel, Switzerland) using a VIIA7 thermal cycling system (Applied Biosystems, Carlsbad, USA). The thermal cycling conditions were 2 min at 95°C, followed by 15 sec at 95°C, 30 sec at 60°C, and 30 sec at 72°C, for a total of 40 cycles. Primers were designed and tested to have a calculated 95%–105% reaction efficiency. For each gene, fluorescent intensity was related to the fluorescent intensity of the reference gene tyrosine 3‐monooxygenase/tryptophan 5‐monooxygenase activation protein, zeta polypeptide (*YWHAZ*). mRNA expression levels of genes from the collagen biosynthesis pathway and other ECM components were analyzed with a custom made microfluidic card‐based low‐density array (Applied Biosystems) and a VIIA7 thermal cycling system, as described previously (Piersma et al. 2015).

### Immunofluorescence

For immunofluorescence of Smad2, cells were washed twice with PBS and fixed with 2% paraformaldehyde (Sigma‐Aldrich) for 10 min. For immunofluorescence of smooth muscle *α*‐actin (*α*SMA), collagen type I, procollagen type I, and collagen type IV, cells were washed twice in PBS and fixed with ice‐cold methanol/acetone (1:1) for 10 min at −20°C. Methanol/acetone fixed cells were first dried and later rehydrated with PBS before use. For all immunofluorescent stainings except collagens type I and IV, fixed cells were permeabilized with 0.5% Triton X‐100 in PBS for 10 min, and incubated with 2.2% bovine serum albumin (BSA) for 30 min. Next, cells were incubated for 1 h with primary antibodies: mouse monoclonal to *α*SMA (Clone 1A4, 0.28 *μ*g/mL; Dako, Glosstrup, Denmark), mouse monoclonal to collagen type I (ab90395, 1 *μ*g/mL; Abcam, Cambridge, United Kingdom), goat polyclonal to procollagen type I (sc‐8782, 2 *μ*g/mL; Santa Cruz Biotechnology, Dallas, TX, USA), or mouse monoclonal to Smad2 (L16D3, 0.5 *μ*g/mL; Cell Signaling Technologies, Leiden, the Netherlands) in PBS containing 2.2% BSA. After three washes with PBS, cells were incubated with fluorescent labeled secondary antibodies.

### Immunoblotting

Cells were lyzed with RIPA buffer (Thermo Fisher Scientific) supplemented with protease inhibitor cocktail (Sigma‐Aldrich) and sonicated. The DC protein assay (Bio‐Rad, Hercules, CA) was used to quantify protein concentrations and equal amounts of protein (20 *μ*g/lane) were subjected to SDS gel electrophoresis on stain‐free TGX mini‐PROTEAN precast gels. Before protein transfer, hydrogels were put under UV to activate stain‐free trihalo components in the gel, and gel images were taken for total protein quantification and normalization as described previously (Ladner et al. 2004). After activation, protein transfer to a nitrocellulose membrane was performed using the semidry Transblot Turbo system (Bio‐Rad). Membranes were blocked in 5% skimmed milk in Tris‐buffered saline + 0.1% Tween 20 and incubated overnight with primary antibodies: mouse monoclonal to *α*SMA (Clone 1A4, 0.28 *μ*g/mL; Dako) and goat polyclonal to collagen type I (sc‐8783, 2 *μ*g/mL; Santa Cruz Biotechnology). Next day, after three washes with TBST, membranes were incubated with goat‐anti‐mouse HRP (P0447, 1 *μ*g/mL; Dako) or rabbit‐anti‐goat HRP (P0049, 0.5 *μ*g/mL; Dako) for 1 h at RT. Protein bands were visualized with chemiluminescence (ECL, Thermo Fisher Scientific) and a ChemiDoc imaging system (Bio‐Rad). Image analysis was performed with ImageJ version 5.1 (Schindelin et al. 2012).

### Collagen lattice contraction assay

After 3 days of stimulation, dermal fibroblasts were seeded in collagen lattices with a final concentration of 2.4 mg/mL rat tail collagen type I (354249; BD, San Jose, CA), 1× PBS, 20 mmol/L HEPES, 5.8 mmol/L NaOH, 50% EMEM complete growth medium, and 5% FBS. Cells were seeded at a concentration of 2 × 10^5^/mL gel. Cells were allowed to prestress the collagen lattice 3 days prior to detachment, while continuing stimulation with either or both TGF‐*β*1 and AA. At time point *t* = 0 min, gels were released from the well rim and allowed to contract. Well plates were scanned at multiple time points on a flatbed scanner. Collagen lattice contraction was calculated using ImageJ version 5.1.

### Transient transfection and luciferase assay

For the measurement of Smad2/3 transcriptional activity, cells were transfected with 2 *μ*g plasmid DNA containing four copies of a Smad‐binding element (SBE4‐luc, Addgene #16495) (Zawel et al., 1998) using Lipofectamine LTX and PLUS reagent (Thermo Fisher Scientific) in bare EMEM. After 24 h, cells were starved for 4 h in EMEM with 0.5% FBS (bare medium) and subsequently stimulated with either bare medium, TGF‐*β*1, AA, or both for 18 h. Cells were lyzed and luciferase activity was detected using a luciferase assay system (E1500; Promega, Leiden, the Netherlands) according to the manufacturer's instructions. The average fold‐change was calculated from three independent experiments and normalized against total protein concentration.

### Statistics

All data were tested with two‐way ANOVA combined with Bonferroni post hoc testing using GraphPad Prism version 7.01 for Windows (GraphPad, La Jolla, CA).

## Results

### Ascorbic acid and TGFβ1 work in synergy with respect to (pro)collagen type I deposition

**Figure 1:**
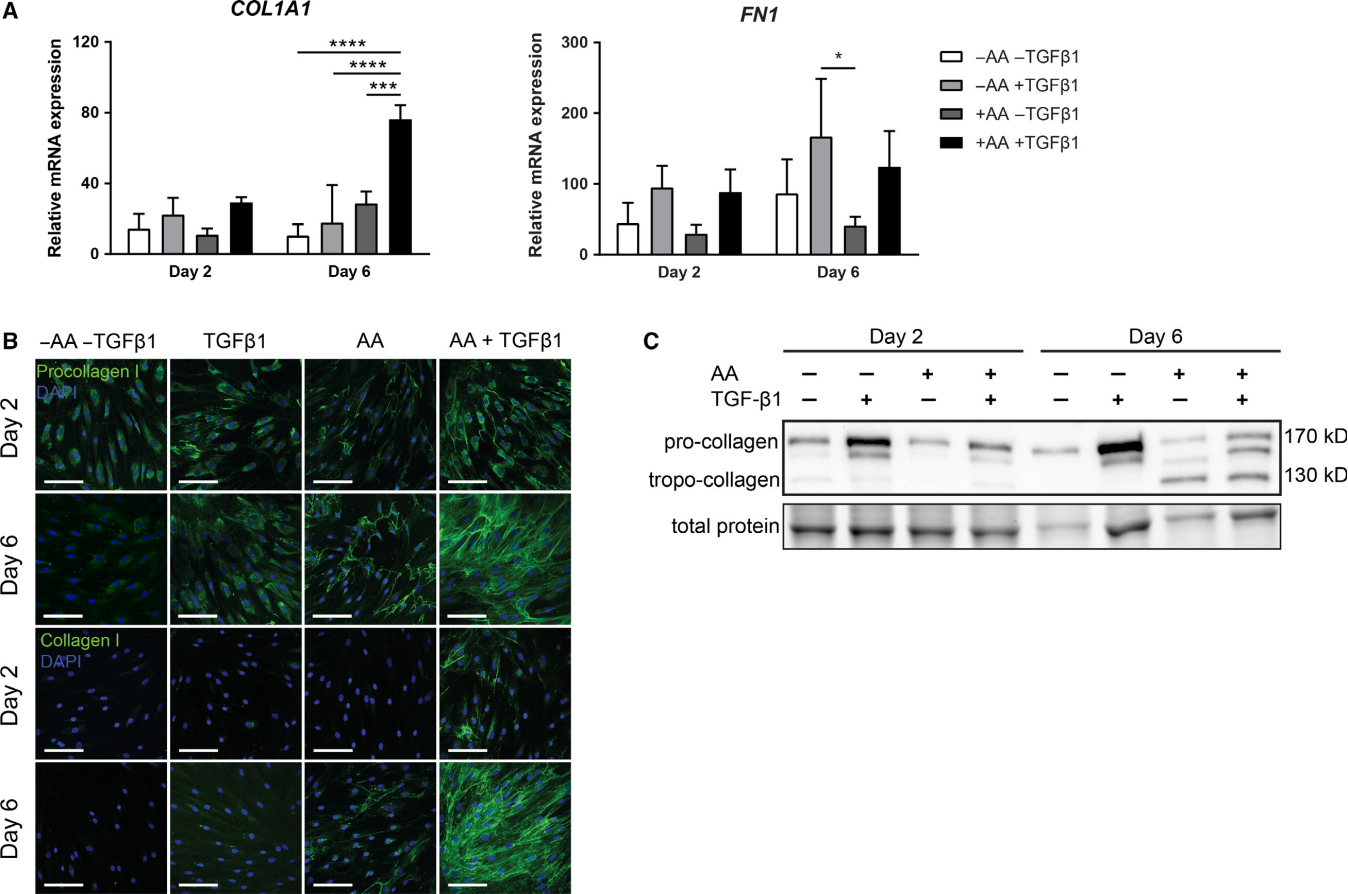
Ascorbic acid synergizes with TGF *β*1 to govern collagen production. (A) Relative mRNA expression of *COL1A1* and *FN1*. Cells were exposed to bare medium or TGF *β*1 for 2 or 6 days with or without addition of AA. *n *= 3 individual experiments. (B) Representative immunofluorescent confocal photomicrographs of procollagen type I and native collagen type I staining on days 2 and 6. Original magnification 630×; scale bar = 100 *μ*m, *n* = 3 individual experiments. (C) Immunoblot on complete cell lysates after 2 and 6 days with an antibody recognizing the *α*1 chain of native and denatured procollagen and collagen type I. Total protein loading control was visualized with trihalo compound which were activated on UV light exposure. A representative part of the total protein blot is shown. *n* = 3 individual experiments. Data are represented as mean ± SD. Two‐way ANOVA with Bonferroni posttest. *P < 0.05; **P < 0.01; ***P < 0.001; ****P < 0.0001. AA, ascorbic acid; kD, kilo Dalton; TGF *β*1, transforming growth factor *β*1.

To determine whether AA affects mRNA expression levels of *COL1A1*, we cultured human dermal fibroblasts in the presence or absence of AA and/or TGF*β*1 for 2 and 6 days. No changes were seen in *COL1A1* mRNA levels at days 2 and 6 when either AA or TGF*β*1 are added. In contrast, higher mRNA levels of *COL1A1* were seen at day 6 when AA and TGF‐*β*1 were added together (Fig. 1A). Another extracellular matrix molecule, fibronectin (FN1), was – in contrast to (pro)collagen type I – not affected by AA (Fig. 1A). We next investigated the presence of procollagen by means of immunofluorescence with an antibody that recognizes to the *α*1(I) N‐propeptide. Intracellular procollagen was present under all conditions, whereas extracellular procollagen was only seen at days 2 and 6 when AA or AA + TGF*β*1 was present (Fig. 1B). Staining with an antibody that recognizes native (triple helical) but not denatured (pro)collagen showed the absence of triple helical (pro)collagen in the absence of AA, whereas both intra‐ and extracellular triple helical (pro)collagen were seen at day 6 when AA was present and at days 2 and 6 when AA + TGF*β*1 was present (Fig. 1B). The immunofluorescence data regarding the amount of procollagen as well as native collagen both confirm that AA and TGF*β*1 work in synergy. The immunofluorescence data were verified with immunoblotting using an antibody recognizing both the native and the denatured triple helical part of the procollagen *α*1(I) chain. It indeed shows the presence of procollagen under all culture conditions, and furthermore a prominent presence of collagen at day 6 when AA or AA + TGF*β*1 were present (Fig. 1C). Since procollagen can only be converted into collagen when it is in a triple helical (native) state, the prominent presence of collagen at day 6 under AA or AA + TGF*β*1 shows that AA is required for procollagen to adapt a triple helical format.

### Ascorbic acid facilitates the TGFβ1‐induced differentiation of fibroblasts into myofibroblasts

**Figure 2:**
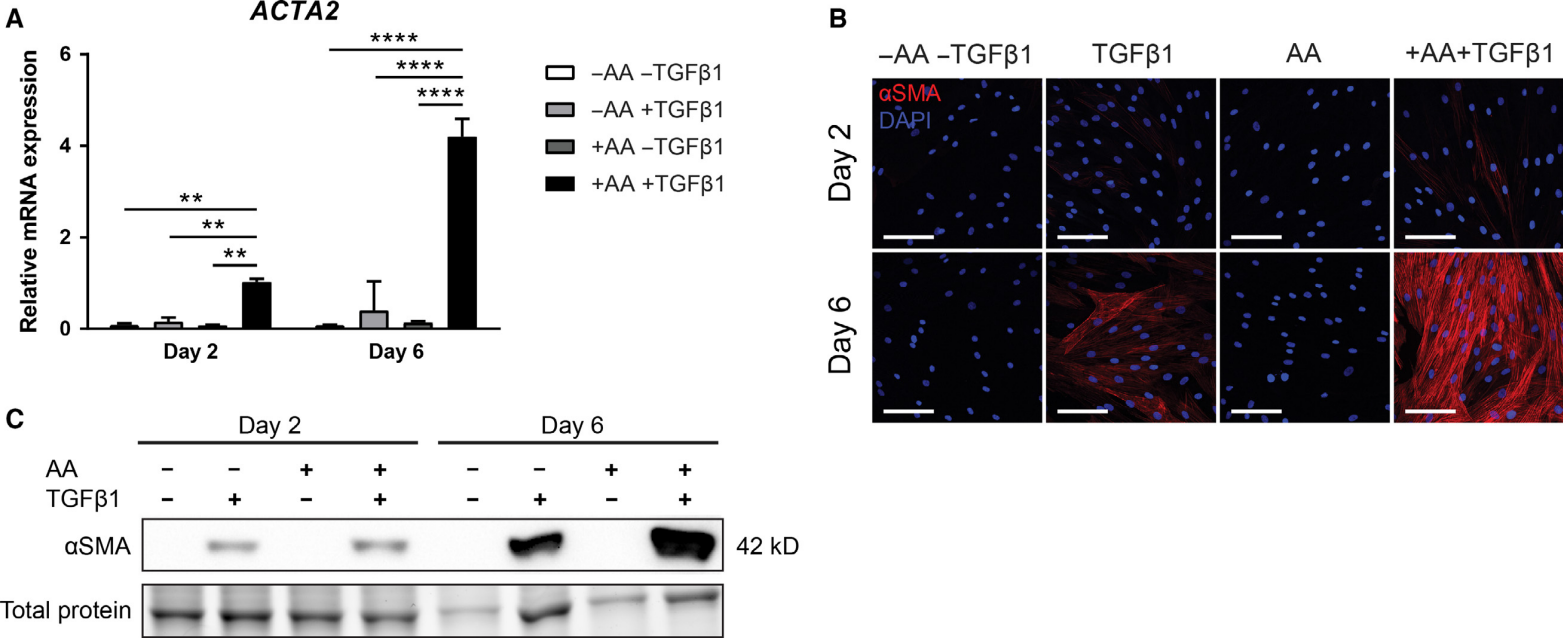
Ascorbic acid amplifies TGF *β*1‐induced *α* SMA expression. (A) Relative mRNA expression of *ACTA2*. Cells were exposed to bare medium or TGF *β*1 for 2 or 6 days with or without addition of AA. *n* = 3 individual experiments. (B) Representative immunofluorescent confocal photomicrographs of *α* SMA staining on days 2 and 6. Nuclei are visualized with DAPI. Original magnification 630×; scale bar = 100 *μ*m. *n* = 3 individual experiments. (C) Immunoblot on complete cell lysates for *α* SMA after 2 and 6 days. Total protein loading control was visualized with trihalo compound which were activated on UV light exposure. A representative part of the total protein blot is shown. *n* = 3 individual experiments. Data are represented as mean ± SD. Two‐way ANOVA with Bonferroni posttest. **P < 0.01; ****P < 0.0001. AA, ascorbic acid; kD, kilo Dalton; *α* SMA,* α* smooth muscle actin; TGF *β*1, transforming growth factor *β*1.

**Figure 3:**
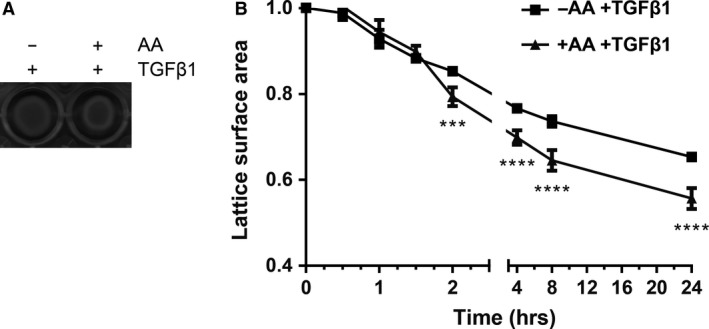
Ascorbic acid promotes TGF *β*1‐induced collagen contraction. (A) Representative photo scan of a 24‐well plate containing a myofibroblast‐populated collagen lattice, exposed to TGF *β*1 and stimulated with or without AA for 72 h. (B) Quantification of A. *n *= 3 individual experiments. Data are represented as mean ± SD. Two‐way ANOVA with Bonferroni posttest. ***P < 0.001; ****P < 0.0001. AA, ascorbic acid; TGF *β*1, transforming growth factor *β*1.

We wondered, since AA and TGF*β*1 work in synergy with respect to mRNA levels of *COL1A1* and protein levels of procollagen and collagen, whether AA is also involved in TGF*β*1‐induced myofibroblast formation. A marker of myofibroblasts is the presence of *α*SMA stress fibers, which are absent in quiescent fibroblasts (Hinz et al. 2001). We first measured mRNA (*ACTA2*) and protein levels of *α*SMA, and found that AA or TGF*β*1 alone did not significantly increase *ACTA2* mRNA levels, but that the combination AA + TGF*β*1 does result in a major increase, both at day 2 and day 6 (Fig. 2A). Staining for *α*SMA showed a modest formation of stress fibers at day 6 under the influence of TGF*β*1, whereas an abundance of stress fibers was observed with the combination AA + TGF*β*1 (Fig. 2B). Incubation with AA alone did not result in the formation of stress fibers. Immunoblots showed elevated levels of *α*SMA at days 2 and 6 in the presence of TGF*β*1 or AA + TGF*β*1, but not with AA alone (Fig. 2C). The highest level was found at day 6 in the presence of AA + TGF*β*1. These data show that AA facilitates the TGF*β*1‐induced myofibroblast formation. Since *α*SMA stress fibers contribute to myofibroblast contractility (Hinz et al. 2001; Subramanian et al. 2004), we assessed the impact of AA combined with TGF*β*1 on myofibroblast contractility with a collagen lattice contraction assay. Combined stimulation with AA and TGF*β*1 indeed resulted in increased contraction in a 3D collagen lattice (Fig. 3A and B).

### AA enhances the expression of COL4A1, CCN2, and DDR1

**Figure 4:**
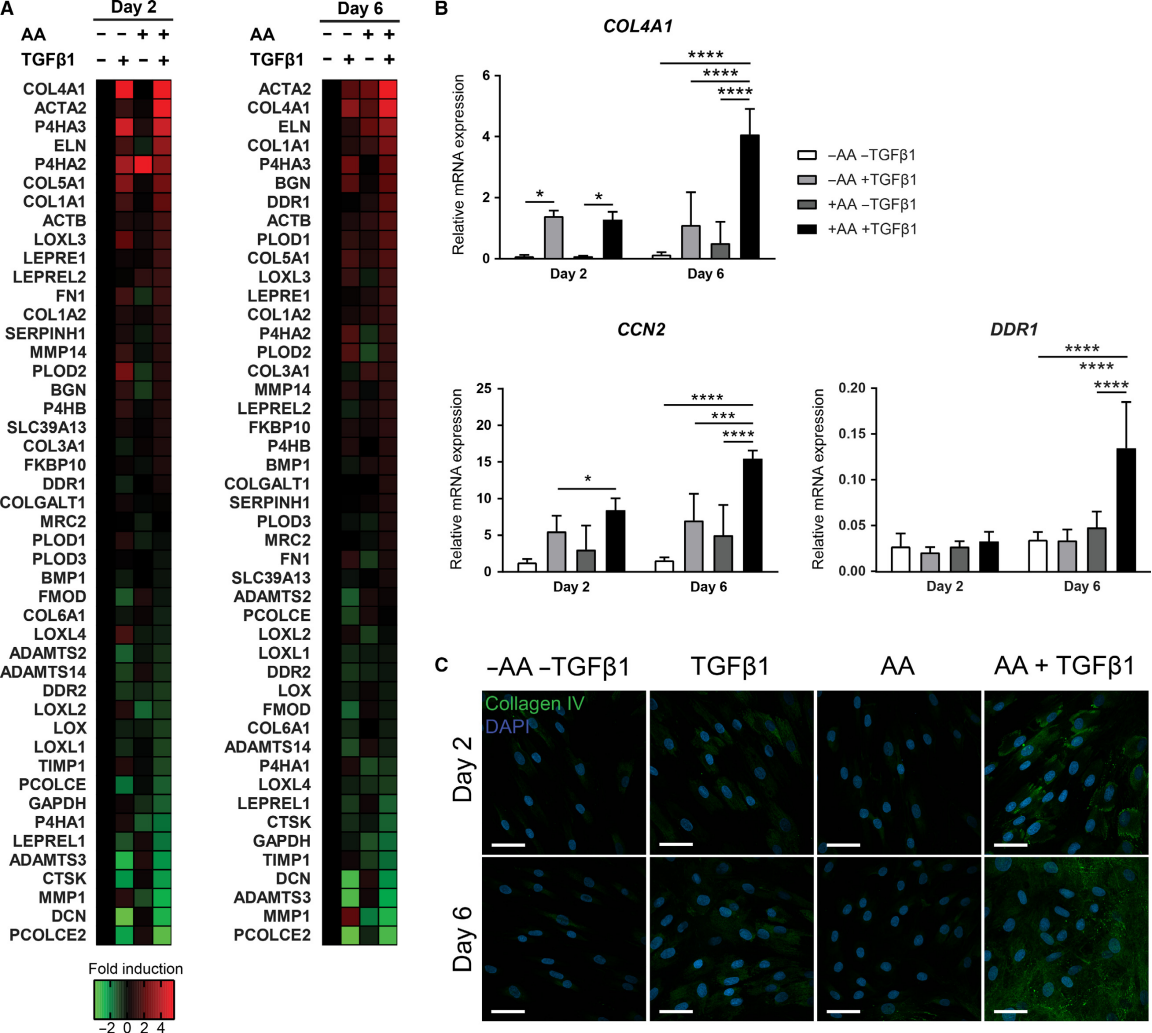
Ascorbic acid synergizes with TGF *β*1 to mediate expression of ECM components. (A) Heat map of a microfluidic card‐based low‐density array‐based mRNA expression. Cells were exposed to bare medium or TGF *β*1 for 2 or 6 days with or without addition of AA. Heat map shows fold induction over bare treatment (‐AA ‐TGF *β*1). *n* = 3 individual experiments. (B) Relative mRNA expression of *COL4A1*,*CCN2*, and *DDR1*. (C) Representative immunofluorescent confocal photomicrographs of collagen type IV staining. *n* = 1 experiments. Original magnification 630×; scale bar = 50 *μ*m. Data are represented as mean ± SD. Two‐way ANOVA with Bonferroni posttest. *P < 0.05; ***P < 0.001; ****P < 0.0001. AA, ascorbic acid; TGF *β*1, transforming growth factor *β*1.

Since AA seems to be involved in the TGF*β*1‐induced myofibroblast phenotype, we investigated whether AA is involved in the expression of other genes known to be upregulated in myofibroblasts. We analyzed the expression of genes coding for various ECM components together with proteins and enzymes involved in collagen synthesis and degradation. Microfluidic card‐based low‐density array analysis revealed that compared to bare medium or AA alone, TGF*β*1 increases the expression of multiple genes, including *COL4A1*,* P4HA2*,* P4HA3*, and *COL5A1* (Fig. 4A). Moreover, combined stimulation with TGF*β*1 and AA lead to further upregulation of *COL4A1* (Fig. 4A and B). The profibrotic gene CCN2 showed a similar upregulation when AA and TGF*β*1 are combined (Fig. 4B). These data suggest that AA increases the expression of some but not all TGF*β*1‐responsive genes. Immunofluorescence analysis confirmed the increase of collagen type IV after TGF*β*1 stimulation and that AA enhances the synthesis of collagen type IV compared to TGF*β*1 alone (Fig. 4C). An example of a gene that is nonresponsive toward TGF*β*1 alone but that is expressed on combined exposure to TGF*β*1 and AA is the collagen receptor DDR1 (Fig. 4A and B).

### Ascorbic acid mediated myofibroblast phenotype switch is Smad2/3 independent

**Figure 5:**
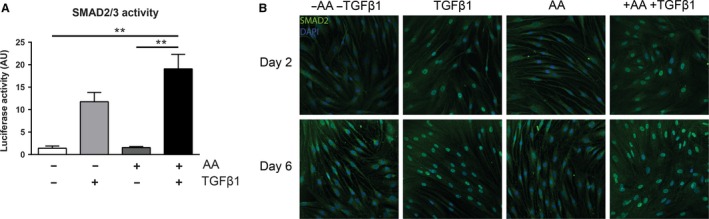
Ascorbic acid does not affect Smad2/3 signaling. (A) Smad‐binding element (SBE) promoter activity luciferase assay. *n* = 3 individual experiments. (B) Representative confocal immunofluorescent photomicrographs of Smad2 staining. Original magnification 630×; scale bar = 50 *μ*m. *n* = 1 experiments. Data are represented as mean ± SD. Two‐way ANOVA with Bonferroni posttest. **P < 0.01. AA, ascorbic acid; AU, arbitrary units; TGF *β*1, transforming growth factor *β*1.

Smad2, together with Smad3, are the major transcriptional effectors of the canonical TGF*β*1 signaling cascade, and have been shown to govern the expression of several collagens and *α*SMA (Evans et al. 2003; Subramanian et al. 2004; Dobaczewski et al. 2010). To investigate the relationship between AA and Smad2/3 transcriptional activity, we transiently transfected fibroblasts with a SBE4‐luc promotor construct containing four copies of a Smad‐binding element in front of a luciferase reporter gene. TGF*β*1 alone increased the transcriptional activity of Smad2/3 compared to bare medium and AA alone (Fig. 5A). However, combined stimulation of TGF*β*1 and AA did not further enhance luciferase activity significantly. Moreover, immunofluorescence analysis revealed that AA addition does not enhance the nuclear accumulation of Smad2 compared to TGF*β*1 alone (Fig. 5B). Thus, whether the effects of AA on the expression of signature myofibroblast genes are dependent of Smad2 and Smad3 remains inconclusive.

## Discussion

We investigated the effect of AA in the presence or absence of TGF*β*1 on collagen deposition at days 2 and 6 in more detail by means of quantitative RT‐PCR and with antibodies directed toward the N‐propeptide of procollagen type I, the native (triple helical) structure of the collagenous part of procollagen type I (thus recognizing only native collagen and procollagen), and an antibody recognizing procollagen and collagen type I in both its native and denatured state. We show that AA alone does not change mRNA levels of *COL1A1* on days 2 and 6 in the absence of TGF*β*1, whereas to our surprise AA resulted in a major increase of *COL1A1* mRNA levels at day 6 in combination with TGF*β*1. At the protein level, procollagen was observed in all experimental conditions (bare medium; TGF*β*1; AA; AA + TGF*β*1) intracellularly, whereas extracellular procollagen was observed only in the presence of AA. In order to discriminate between procollagen in a helical or nonhelical form, we used an antibody that recognizes only the helical form of procollagen and collagen. It turned out that native procollagen (or native collagen) was barely present under control or TGF*β*1 conditions, but it was clearly present in the presence of AA. The presence of extracellular procollagen or extracellular native collagen/procollagen was most obvious when AA was combined with TGF*β*1.

From the protein data one can conclude that, although procollagen is present in all conditions, the procollagen is only present in its triple helical form when AA is present. We did not observe extracellular procollagen in the absence of AA, indicating that the non‐native procollagen is not excreted by the cell and/or is immediately degraded in the extracellular space. The immunoblot revealed that at day 6 the non‐native procollagen was not processed into collagen in the absence of AA (i.e., the N‐propeptides and C‐propeptides were not cleaved off), whereas collagen was seen in the presence of AA. Indeed, cleavage of the N‐propeptides occurs only when the procollagen is in its native state (Tuderman et al. 1978; Tanzawa et al. 1985; Prockop et al. 1998). The native state is facilitated by the presence of Hyp (Jimenez et al. 1973). Absence of AA results in a severe underhydroxylation of proline residues, since AA is a cofactor for prolyl hydroxylase (Myllyharju 2008).

It should be stressed that most antibodies toward collagen type I react with both native and denatured collagen proteins. Studies carried out with human fibroblasts in the absence of AA will detect collagen with such antibodies, but by far the majority of this “collagen” actually represents non‐native procollagen. Not knowing this will clearly lead to unreliable conclusions and this can unfortunately be readily observed in the existing literature.

It is well‐known that TGF*β*1 promotes the differentiation of fibroblasts into myofibroblasts (Hao et al. 1999; Evans et al. 2003; Leask and Abraham 2004; Dobaczewski et al. 2010), a process that can be followed by measuring *α*SMA (encoded by *ACTA2*). Much to our surprise, we observed that the differentiation of fibroblasts into myofibroblasts is highly facilitated by AA, as shown by the dramatically increased *ACTA2* mRNA levels when TGF*β*1 was combined with AA. mRNA levels of *ACTA2* were not increased in the presence of AA alone, so there is a clear synergy between AA and TGF*β*1. This was also obvious at the protein level: staining for *α*SMA stress fibers revealed much more myofibroblasts at day 6 compared to AA or TGF*β*1 alone, which indeed resulted in an increased contraction of a 3D collagen lattice. Thus, AA works in synergy with TGF*β*1, facilitating the profibrotic properties of TGF*β*1. However, the heat map of the low‐density array showed that not all TGF*β*1‐responsive genes were additionally upregulated by AA, suggesting that the action of AA is not regulated via Smad2/3, being the canonical TGF*β*1 pathway. Indeed, we observed that AA did not enhance the nuclear translocation of Smad2, and no significant increased activity was observed with a luciferase reporter containing four copies of a Smad‐binding element.

The heat map shows that the addition of AA alone results in a change in the expression pattern of only a few genes, but that AA in combination with TGF*β*1 is involved in the general enhancement of the myofibroblast expression profile in dermal fibroblasts. However, how exactly AA amplifies the TGF*β*1‐induced myofibroblast phenotype remains elusive. We speculate that this is likely due to the function of AA in epigenetics (Monfort and Wutz 2013; Camarena and Wang 2016). Several studies highlighted that AA is involved in the process of active demethylation of cytosine (5mC), mediated by the Ten‐eleven translocation (Tet) methylcytosine dioxygenases enzymes Tet1, Tet2, and Tet3 (Tahiliani et al. 2009; Minor et al. 2013). Conventionally, 5mC is regarded as mark for the transcriptionally repressed chromatin, and DNA methylation of lineage‐specific loci governs cellular differentiation programs (Kohli and Zhang 2013). Similar to the prolyl hydroxylases P3H and P4H, AA acts as electron donor for Tets and reduces Fe^3+^ to Fe^2+^. Moreover, it is thought that AA also acts as cofactor for the Jumonji C‐domain containing histone demethylases (Tsukada et al. 2006; Wang et al. 2011). Methylation of histones is described as another tier of chromatin remodeling, which is associated with either activation of repression of transcription (Greer and Shi 2012). The importance of AA in determining the epigenetic landscape has also emerged in the reprogramming of induced pluripotent stem cells, which are unable to be fully reprogrammed in the absence of AA (Wang et al. 2011).

In conclusion, we have not only shown why AA is crucial in the process of collagen production, but also that AA facilitates the TGF*β*1‐induced adoption of a myofibroblast phenotype. Thus, AA is involved in fibrotic processes at multiple levels. Finally, we speculate that AA induces epigenetic changes, thereby regulating expression of multiple myofibroblast‐related genes.

## Conflict of Interest

None declared.
